# Effects of different drying methods and ascorbic acid pretreatment on carotenoids and polyphenols of papaya fruit in Ethiopia

**DOI:** 10.1002/fsn3.2324

**Published:** 2021-05-04

**Authors:** Masresha Minuye, Paulos Getachew, Arnaud Laillou, Stanley Chitekwe, Kaleab Baye

**Affiliations:** ^1^ Center for Food Science and Nutrition Addis Ababa University Addis Ababa Ethiopia; ^2^ United Nations Children’s Fund (UNICEF) Addis Ababa Ethiopia

**Keywords:** diets, food systems, fruits, green technologies, solar drying, vitamin A

## Abstract

Frequent consumption of fruits can prevent nutrient deficiencies and promote health. However, the perishability and unaffordability of fruits had led to very low levels of fruit consumption in low‐ and middle‐income countries (LMICs). The objective of this study was to evaluate the retention of nutrients and bioactive compounds of papaya fruit (*Carica papaya* L) with/without ascorbic acid pretreatment and drying under different techniques, to then estimate the vitamin A intakes for vulnerable population. Yellow pulp ripped papaya fruits (i.e., >75% level of skin color/stage level 4) (*n* = 14), with and without ascorbic acid pretreatment were dried using (a) solar drying: open‐air, tray driers, and glasshouse; (b) refractance window drying; (c) oven drying; and (d) freeze‐drying (control). The fresh fruit had high moisture content (87%) and an acidic pH. The dried papaya had a water activity of 0.5–0.6. The highest TPC, TFC, total carotenoids, and ß‐carotene were found in freeze‐dried papaya samples, followed by refractance window, and solar glass house (*p* < .05). The highest retention in total carotenoids (81.5%) and ß‐carotene (61.9%) relative to freeze‐drying was for the refractance‐window; 25 g of dried papaya could contribute to 38% of the retinol equivalents’ requirement for young children. Ascorbic acid pretreatment increased the retention of total carotenoids, ß‐carotene, TPC, and TFC (*p* < .05) by (6–11)%, (8–34)%, (7–58)%, and (6–30)%, respectively, for all the drying methods. Refractance window and solar glass house drying can improve diets and constitute a promising food systems’ intervention that can increase year‐round availability, accessibility, and affordability of vitamin A‐rich fruits like papaya.

## Graphical abstract

This study evaluated the retention of total polyphenols, flavonoids, ß‐carotene contents of papaya fruit with ascorbic acid pretreatment and dried under different techniques of solar, refractance window, and oven drying. Refractance window and solar glass house drying can constitute a promising food systems to increase year‐round availability of vitamin A‐rich fruits like papaya.

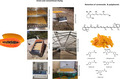

## 1 BACKGROUND

One in three people worldwide is affected by one or more forms of malnutrition. About 800 million are undernourished, 1.9 billion adults are overweight or obese (WHO, 2017), and two billion people worldwide are anemic and suffer from micronutrient deficiencies (Camaschella, 2015). The underlying driver of all forms of malnutrition is unhealthy diet (Pradeilles et al., 2019). Over three billion people cannot afford even the cheapest healthy diet (SOFI, 2020). Poor nutrition is now considered the leading cause of morbidity and mortality putting people at a greater risk than unsafe sex, tobacco, drug, and alcohol use combined. Although what constitutes a healthy diet is context‐dependent, there is consolidated evidence that diverse diets with consumption of nutrient‐dense foods like whole grains, legumes, nuts, fruits, and vegetables (FVs) are associated with healthier outcomes (Herforth et al., 2019). Despite irrefutable evidence of the benefits of frequent consumption of fruits, their consumption is very low in low‐ and middle‐income countries (Miller et al., 2016). A recent analysis of children's diet in 49 LMICs revealed that very few consume vitamin A‐rich FVs (Baye & Kennedy, 2020). In Ethiopia, for example, the average household consumed 45 kg of FVs per adult equivalent (Worku et al., 2017). This level is among the lowest in sub‐Saharan Africa and is far from meeting the WHO recommendation of 146 kg per year (Ruel et al., 2005). A recent review has also shown that only about 2.4% adults meet the WHO recommendation of five servings of fruits and/or vegetables per day, leading to increased risk of nutrient deficiencies (e.g., marginal or clinical vitamin A status) and chronic diseases (Baye & Hirvonen, 2020). The highest (~11%–12%) disability‐adjusted life years (DALYs) and years of life lost (YLLs) were related to this low consumption of fruits (Melaku et al., 2016).

Despite sustained nutrition education through the health system, consumption of fruits has remained very low (Baye & Hirvonen, 2020; Baye et al., 2019) and the reach of interventions like vitamin A supplementation has been inadequate (Laillou et al., 2020). The high price of fruits, their perishability, and seasonality are among the underlying factors influencing consumption of fruits (Baye & Hirvonen, 2020). Although this is recognized, little or no food systems’ innovation has been put in place to reduce the price of FVs by decreasing loss and reducing transaction costs related to transportation, as well as increase the safety and shelf‐life of fruits. This is particularly true in settings where access to electricity and cold chain is a serious challenge. In such settings, solar drying can hold promise of green technology being environmentally friendly and requiring less sophisticated infrastructure, but the nutritional impact of various types of solar drying technologies remains largely unknown. This is unfortunate as such information is important to design much‐needed food systems interventions that promote and enable households to consume fruits more frequently, while keeping the loss of nutrients, taste, and color changes minimal. Mohammed et al., (2020) reported that the nutritional values of mango and pineapples could be retained by comparing five solar drying methods (open sun drying, black‐cloth shade, white cloth shade, a conventional solar dryer, and a newly improved solar dryer); however, their experiment was not controlled. According to Abrol et al. (2014), solar tunnel drying of mango, banana, and papaya showed reduction in vitamin C concentrations. The effect of drying on total polyphenol and antioxidants of the leaves of papaya was also recently evaluated (Yap et al., 2020). However, a comprehensive evaluation of solar drying technologies relative to more common drying methods such as oven and freeze‐drying remains scant. Some of the studies conducted on the influence of solar drying are on different fruits and vegetables.

This study aimed to bridge this gap by evaluating the nutrient retention (total carotenoids and ß‐carotene), total polyphenols, and total flavonoids content of papaya fruit (*Carica papaya* L) with/without ascorbic acid pretreatment and under different drying techniques, including (a) solar drying (open‐air, tray driers, and glass house); (b) refractance drying; (c) oven drying; and (d) freeze‐drying (control). The contribution of dried papaya to vitamin A requirements of children and adults was estimated.

## 2 MATERIALS AND METHODS

### 2.1 Sample preparation

Samples of yellow papaya (*Carica papaya* L) fruit (10–14 for each replication) were brought from the market and were allowed to uniformly ripe until 75% of the skin color changed to yellow. The ripening of the papaya was further evaluated by analyzing the total soluble solids (TSS) and pH. TSS was measured using a portable digital refractometer (Chincan, China (Mainland)), and pH was measured using a portable pH meter (baoshishan, China). Peeling and slicing of the papaya were conducted by hand. The ripped papaya was sliced into cubes of a diameter of 5–6 mm, and was divided into two portions, where half was treated by soaking in ascorbic acid solution (34 g prepared in 1 L water), and the other half was left untreated. The ascorbic acid treatment was according to Kendall and Sofos, (2007). The uniformity of the slices was checked using digital caliper (kotapro, China). Completely randomized experimental design (CRD) was applied, and the experiments were run in triplicate, yielding a total sample of 42.

### 2.2 Drying methods

Apart from the freeze and oven driers, all the others were locally adapted technologies. A picture showing the driers is presented in the supplement (Figure S1).

*Open solar drying* (direct) was conducted by laying the sliced papaya on a plastic‐covered wire mesh. The drying was based on direct exposure to the sun.

*Solar‐tray drying* (indirect) was conducted on a drier that has inclined tray holders with a black sheet background for absorption of solar rays. The tray holders are then covered by a glass. *Glass house* *(green house)* *solar drying:* This is a green house whose walls and roof are made from glass.

*Refractance window water drying:* This drying works on the principle of heat transmittance from boiling water.

*Oven drying* was conducted in a ventilated laboratory oven (DHG‐9123A Zenith Lab, China).

*Freeze‐drying* was conducted using a laboratory scale lyophilizer (freeze dryer).

The retention of nutrients was calculated relative to freeze‐drying (Scientz‐10N, China), which is known to yield maximal retention of nutrients and bioactive compounds (Marques et al., 2006).

### 2.3 Weight loss and yield

The weight of the papaya fruit right after harvest (day 1) till ripening (days 4–6) was recorded. After ripening, the weight losses related to peeling, removal of seeds, slicing, and drying were recorded to calculate the average yields (%).

### 2.4 Analytical procedures

#### 2.4.1 Moisture and water activity

The moisture content was measured before and after drying by oven drying at 105°C to constant weight (protocol no: AOAC. 925.10; AOAC International, 2007).

Water activity (a_w_) of the dried papaya fruit was measured at 25°C using a water activity measurement device (Wert‐Messer, Germany).

#### 2.4.2 Temperature and relative humidity

Temperature and relative humidity during drying were measured using a portable device (Vici 288B‐CTH, Guangdong, China).

#### 2.4.3 Total carotenoids

Total carotenoids were performed following methods described by the Harvest Plus handbook for carotenoid analyses (Rodriguez‐Amaya & Kimura, 2004). Briefly, 5 g of dried papaya samples was ground in 40 ml acetone using mortar and pestle until the mix became colorless. The extract was vacuum‐filtered using a Buchner funnel, partitioned with 60 ml of petroleum ether, and then, each fraction was washed with distilled water for complete acetone removal. The extracts were made up to a volume of 50 ml with petroleum ether. All of the procedures were performed in dim light, and the absorption of the extract was read at 452 nm using a UV spectrophotometer (Shimadzu ‐UV‐1800, Japan). The total carotenoids concentration was calculated by applying the following formula:

(1) Total Carotenoid(μg/g)=[A×V(ml)×104]/[A1cm1%×sample weight(g)]

Where: A = absorbance; V = total volume of extract (50 ml); A1%1cm = molar absorption coefficient of β‐carotene in petroleum ether (2,592).

#### 2.4.4 β‐carotenes

For the extraction of β‐carotene, the procedure outlined in AOAC Official Method 941.15 was followed (AOAC, 2006). Briefly, 5 g of dried papaya sample was mixed with 40 ml acetone, 60 ml petroleum ether, and 0.1 g magnesium carbonate. The extract was filtered using suction pump and decanted in a separator funnel. The residue was washed with 25 ml acetone and then with 25 ml petroleum ether, and the extracts were combined and evaporated to dryness. The residue was further dissolved in acetone. The extract solution was made up to 5 ml using acetone, filtered through a syringe (0.45 µm) and then injected into the high‐performance liquid chromatography (HPLC; Shimadzu, Japan, Kyoto) with a C18 column (5 µm C18, 4.6 × 250 mm, USA) equipped with a UV detector. The following conditions were set for the HPLC reading: mobile phase: acetonitrile 60%, methanol (30%) and acetone (10%); injection volume*:* 20 µl; flow rate: 2 ml/min; wavelength: 450 nm. The β‐carotene concentration (μg/g) was calculated using the following formula (2).

(2) $$\beta \;\text{- carotene}\left(\mu g/g\right)=\frac{A_{X}\times C_{S}\left(\mu g/\text{ml}\right)\times \text{total volume of extract (ml)}}{\left[A_{S}\times \text{sample weight}\left(g\right)\right]}$$

Where A_x_ = peak area of carotenoid of sample; C_s_ = concentration of the standard; A_s_ = peak area of the standard.

The β‐carotene (μg) was converted to retinol equivalent (RE) by multiplying by 0.167 and was compared to estimated needs from complementary foods (Lutter & Dewey, 2003) and daily requirements for lactating and pregnant women (WHO, 2004).

#### 2.4.5 Bioactive compounds

##### Total phenolic content

The total phenolic compounds of extracts were determined with the folin–ciocalteu method (Singleton et al., 1999). Crude extract of 100 µl (10 mg/ml) was mixed with 0.2 ml folin–ciocalteu reagent, 2 ml purified water, and 2 ml of 7.5% Na_2_CO_3_. The mixture was incubated for 2 hr at room temperature, before reading the absorbance at 765 nm against gallic acid standard, using UV‐Vis spectrophotometer (Genesys‐10 UV, USA). The total phenolic compounds concentration was calculated and expressed as Gallic Acid Equivalent (GAE), as follows:

(3) $$C\left(\text{mg}/\text{g, in GAE}\right)=C_{1}*V/m$$

Where C = total phenolic concentration in mg GAE/g sample, C_1_ = concentration of Gallic acid established from the calibration curve in mg/ml, V = volume of extract in ml; and m = the weight of the papaya extract in g.

##### Total flavonoids

The total flavonoids concentration was determined using the spectrophotometric method described by Quettier‐Deleu et al., (2000). The sample was first extracted in acidified 80% methanol (10 mg/ml), and then, the extract (2 ml) was mixed with equal amounts of 2% AlCl_3_ solution in methanol. The mixed solution was incubated for 1 hr at room temperature and the absorbance was read at 415 nm against a quercetin standard, using a UV‐Vis spectrophotometer. The concentration of flavonoids was expressed as quercetin equivalent (mg of QAE /g of sample), using the following equation:

(4) C(mg/g, inQE)=C1∗V/m

Where C = total flavonoid content in mg/g, in QE (quercetin equivalent), C1 = concentration of quercetin established from the calibration curve in mg/ml, V = volume of extract in ml, and m = the weight of the papaya extract in g.

### 2.5 Statistical analyses

Values are presented as means ± standard deviations (*SD*). Comparisons of means between ascorbic acid treated and untreated samples were performed using independent *t* test. Comparisons by drying methods were performed using analysis of variance (ANOVA), with Tukey‐HSD post hoc test. *p*‐values <0.05 were considered statistically significant. All analyses were performed using SPSS software version 22.0 (SPSS Inc. Illinois, USA).

## 3 RESULTS AND DISCUSSION

### 3.1 Characteristics of the fresh papaya and effect of drying methods on moisture and water activity of dried papaya fruit

In this study, we have collected fresh yellow papaya that was allowed to uniformly ripe. The characteristics of the fresh papaya fruits is presented in Table 1. The papaya fruit had high moisture content (87%) and a slightly acidic pH. Part of the water was lost during the ripening process and the TSS values (10.3%–11.2%) indicated that the papaya was ripped at a physiological maturity level 4 (75% yellow), making it ready for consumption (Barragán‐Iglesias et al., 2018). The highest drying time was for oven drying, which took 20–24 hr to reach a dry matter (DM) of 90% (Table 2). The open (direct)‐, tray (indirect), and glass house solar drying procedures all took 16–18 hr to dry. The solar radiation heats up the slices as well as the surrounding air and thus increases the rate of water evaporation. This is usually done at an average atmospheric air temperature of 28–40°C with relative humidity of less than 60%. In fact, a longer time is expected for solar drying due to fluctuating sun‐light (ambient temperature) than oven drying (Babu et al., 2018). In contrast, the longer time taken for the oven drying might be due to higher load of papaya slices. In contrast, refractance window drying only took 2 hr for drying to 90% DM. Indeed, in the refractance window, three modes of heat transfer (conduction, convection, and radiation) occur at the same time (Raghavi et al., 2018); hence, accelerating the drying process. A water activity between 0.5 and 0.6 was achieved for all the dryings. With such low levels of water activity, the growth of pathogenic and spoilage microorganisms is not favored (Labuza & Rahman, 2007). However, enzymatic and nonenzymatic browning as well as (auto‐) oxidation reactions are likely and can have adverse effects on bioactive compounds like ß‐carotene and polyphenols (Udomkun et al., 2016). Future studies should evaluate the shelf stability of the dried products.

**TABLE 1:** Selected characteristics of the fresh papaya

Parameter	Mean ± *SD*
Moisture (%)	87.2 ± 1.6
pH	5.3 ± 0.1
TSS (^0^Brix)	10.3 ± 0.5
Fresh weight (g)	
Harvest	1,407 ± 260
After ripening	1,292 ± 256

Note

Data are expressed as mean ± *SD* (*n =* *14*). TSS, total soluble solids

**TABLE 2:** Drying time, moisture, and water activity of dried papaya fruit by drying type

Drying	Drying time (h)	Moisture (%)	Water activity
Oven	20–24	9.7 ± 0.4	0.57 ± 0.01
Solar‐open	16–18	12.3 ± 1.4	0.57 ± 0.01
Solar‐tray	16–18	12.8 ± 0.9	0.56 ± 0.01
Solar glass house	16–18	11.3 ± 0.5	0.57 ± 0.01
Refractance window	2	10.3 ± 0.7	0.56 ± 0.01
Freeze‐drying	72	13.3 ± 0.3	0.55 ± 0.01

Note

Data are expressed as mean ± *SD* (*n =* *3*).

### 3.2 Total phenolics, flavonoids, and carotenoids concentration

**FIGURE 1:**
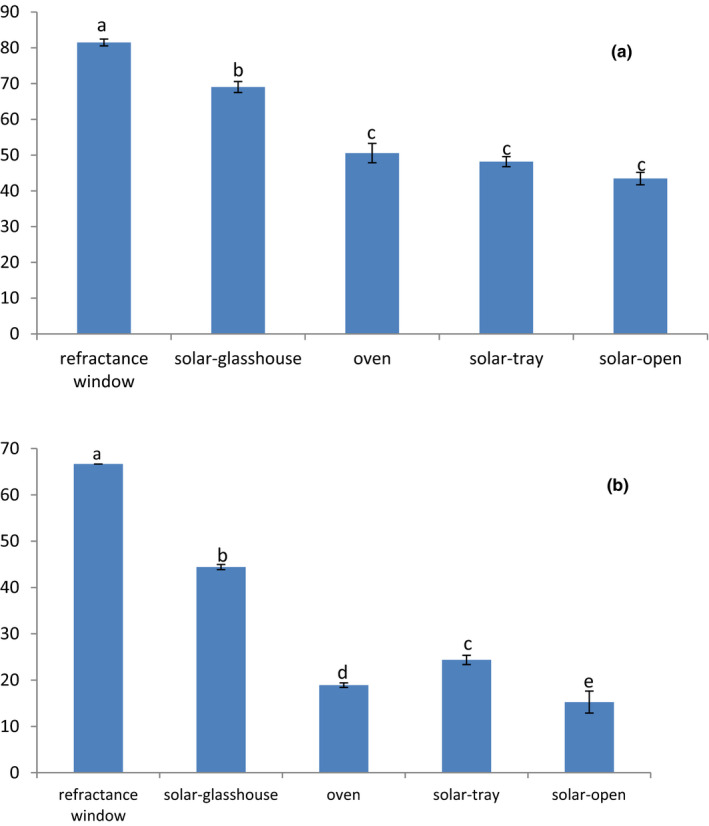
Retention (%) of total carotenoid (a) and beta‐carotene (b) relative to freeze‐dried papaya

Table 3 presents the polyphenol and carotenoid contents in dried papaya obtained under different drying procedures. Not surprisingly, the highest TPC, TFC, total carotenoids, and ß‐carotene were found in freeze‐dried papaya samples, followed by refractance window, and solar glass house. The high nutrient retention in freeze‐dried samples is not surprising as it has been reported to be as one of the best drying method to minimize loss of nutrients and bioactive compounds (Marques et al., 2006; Saini et al., 2014). However, freeze‐drying is operationally expensive and thus not cost‐effective, particularly in LMIC settings (Ratti, 2001). Consequently, drying methods that perform as close as freeze‐drying and environmentally friendly are needed. Figure 1 presents the retention of total carotenoids and beta carotene relative to freeze‐dried samples (control). In line with earlier reports from drying of different fruits, refractance window drying had the best retention, followed by solar glass house (Bernaert et al., 2019; Shende & Datta, 2019). More than 80% of the total carotenoids and 60% of the ß‐carotene were retained after refractance window drying. In contrast, the lowest retentions of total carotenoids (<50%) and beta‐carotene (<40%) were observed for oven, solar‐tray, and open solar drying (Figure 1). Indeed, solar glass house and refractance window drying avoid direct contact of the papaya with heat and light. Previous studies have shown that solar glass house leads to better retention and safety than open‐air drying (Singh et al., 2018).

**TABLE 3:** Total phenolics, flavonoids, carotenoids, and beta‐carotene concentration by drying methods

Drying type	TPC (mg/g GAE)	TFC (mg/g QE)	Total carotenoids (µg/g)	ß‐carotene (µg/g)
Oven	14.78 ± 1.25^c^	1.33 ± 0.06^c^	52.46 ± 2.81^b^	6.40 ± 0.16^b^
Solar glass house	12.01 ± 0.62^bc^	1.95 ± 0.64^c^	71.62 ± 1.60^c^	15.02 ± 0.19^d^
Solar‐open	6.60 ± 0.10^a^	0.80 ± 0.00^a^	45.10 ± 1.80^a^	5.16 ± 0.80^a^
Solar‐tray	11.05 ± 0.60^b^	1.06 ± 0.04^b^	49.97 ± 1.45^b^	8.24 ± 0.34^c^
Refractance window	13.40 ± 1.86^bc^	1.38 ± 0.02^c^	84.55 ± 1.00^d^	22.55 ± 0.01^e^
Freeze‐drying	41.57 ± 2.50^d^	1.89 ± 0.13^d^	103.74 ± 1.11^e^	33.66 ± 0.17^f^

Note

Data are expressed as mean ± *SD* (*n =* *3*). Mean values within the same column with different superscripts are significantly different at *p* < .05.

Abbreviations: GAE, Gallic Acid Equivalent; QE, Quercetin Equivalent; TFC, total flavonoid concentration; TPC, total phenolic concentration.

Refractance window drying belongs to the fourth generation driers and has improved features over previous generations (Bernaert et al., 2019). Drying of the food material happens evenly over a thin infrared transparent material resting over the surface of water that is heated. Thermal energy carried by circulating water transmits heat through the film to the food material by conduction and radiation (Singh et al., 2018). The nutrient retention of foods dried with refractance window is higher than other more common drying systems (e.g., spray drying) and is much more energy efficient (Moses et al., 2014). The energy efficiency of the refractance window drying is three times than spray drying and forty‐fold higher than freeze‐drying (Bernaert et al., 2019).

### 3.3 Effect of ascorbic acid pretreatment on the retention of polyphenols and carotenoids

Table 4 presents the carotenoid and polyphenol retention relative to freeze‐drying. Ascorbic acid pretreatment significantly increased the retention of total carotenoids, ß‐carotenes, TPC, and TFC (*p* < .05). The pretreatment has for example allowed more than 90% of the total carotenoids and beta‐carotenes to be retained in the case of refractance window. This is in line with previous studies showing higher retention in ascorbic acid‐treated mango (Guiamba et al., 2016). Indeed, the ascorbic acid treatment can slow oxidation reactions and prevent browning (Osae et al., 2020).

**TABLE 4:** Effect of ascorbic acid pretreatment on the retention of polyphenols and carotenoids

Drying	Ascorbic acid treatment		*p*‐value
	Untreated (%)	Treated (%)	
Total carotenoids			
Refractance window	81.50 ± 0.96	89.60 ± 2.04	0.003
Solar glass house	69.00 ± 1.54	76.77 ± 2.18	0.007
Solar‐tray	48.20 ± 1.40	58.50 ± 0.78	<0.001
Solar‐open	43.43 ± 1.70	49.63 ± 0.80	0.005
Oven	50.57 ± 2.75	61.37 ± 2.27	<0.001
ß‐carotene			
Refractance window	66.99 ± 0.01	91.70 ± 0.15	<0.001
Solar glass house	44.62 ± 0.55	78.74 ± 4.16	<0.001
Solar‐tray	24.47 ± 1.02	46.66 ± 1.57	0.01
Solar‐open	15.31 ± 2.38	23.42 ± 1.84	0.008
Oven	19.00 ± 0.49	37.79 ± 4.85	0.001
Total phenolic content (TPC)			
Refractance window	32.23 ± 4.47	86.55 ± 2.51	<0.001
Solar glass house	28.74 ± 1.53	86.55 ± 2.35	<0.001
Solar‐tray	26.58 ± 2.52	74.09 ± 6.23	<0.001
Solar‐open	15.78 ± 0.29	22.59 ± 1.52	0.002
Oven	35.56 ± 3.00	89.70 ± 2.29	<0.001
Total Flavonoid content (TFC)			
Refractance window	72.97 ± 0.81	71.73 ± 0.31	0.073
Solar glass house	67.84 ± 1.91	73.85 ± 3.98	0.077
Solar‐tray	56.01 ± 2.14	86.04 ± 3.01	<0.001
Solar‐open	44.52 ± 1.91	59.72 ± 4.90	0.007
Oven	70.67 ± 3.24	89.93 ± 5.23	<0.001

Note

*p*‐values are from comparison of means between untreated and treated using independent *t* test.

### 3.4 Dried papaya contribution to vitamin A requirements

Table 5 presents the contribution of the dried papaya to the vitamin A requirements if added to complementary foods (Lutter & Dewey, 2003). Just 25 g of dried papaya can significantly contribute to vitamin A requirements if added to complementary foods: the highest contribution of retinol was for refractance window drying (38%), followed by solar glass house (25%). For pregnant and lactating women, about 100 g of dried papaya will be required to cover 20%–25% or the daily requirements (Table 5). However, the benefits of carotenoids are far beyond the contribution to vitamin A intake, and relate to their anti‐oxidative properties that confer various health advantages (Eggersdorfer & Wyss, 2018).

**TABLE 5:** Contribution of dried papaya to vitamin A requirements from complementary foods

Drying	Vitamin A (µg RE/100)	% contribution of requirements (in RE)^a^		
		Children 6–23 months	Pregnant women	Lactating women
		25 g	50 g	
Oven	106.7 ± 2.7	11	14	12
Solar glass house	250.3 ± 3.2	25	34	28
Solar‐open	86.0 ± 13.3	9	12	10
Solar‐tray	137.3 ± 5.7	14	19	15
Refractance window	375.8 ± 0.2	38	51	42
Freeze‐drying	561.0 ± 2.8	56	76	62

^a^ RE, retinol equivalent; CF, complementary food; Requirements from complementary foods for children 6–23 months (250 RE) as estimated by Lutter and Dewey (2003); for pregnant and lactating women the estimated daily requirement is 370 and 450 µg RE, respectively (WHO, 2004).

The present study comprehensively evaluates different drying methods that are environmentally friendly on the retention of nutrients and bioactive compounds relative to freeze‐drying. Retention values relative to freeze‐drying, although close to the expected true value, may still lead to an underestimation. The ß‐carotene values may be underestimated as some of the drying processes can lead to trans‐cis isomerization that may not be well differentiated using a C18 column. Although we have evaluated the effect of the dryings on TPC and TFC, more detailed evaluation of the different subtypes of polyphenols is needed.

## 4 CONCLUSION

The present study illustrates that window refractance drying and solar glass house lead to superior quality dried papaya products than those dried with oven, solar open, and solar‐tray drying. Treatment with ascorbic acid led to superior retention than in untreated dried papaya. Promoting the drying of papaya with environmental friendly drying methods like solar drying can extend the shelf‐life and maintain nutritional and bioactive compounds, which in‐turn could increase the availability, accessibility, and affordability of fruits like papaya. Dried papaya can easily be integrated into diets and can significantly contribute to meeting vitamin A requirements. Refractance window and solar glass house drying can constitute a promising food systems’ intervention that can improve year‐round availability, accessibility, and affordability of vitamin A‐rich fruits like papaya.

## CONFLICT OF INTEREST

The authors declare no conflict of interest.

## Supporting information

Fig S1‐S2

## References

[fsn32324-bib-0001] Abrol, G. S. , Vaidya, D. , Sharma, A. , & Sharma, S. (2014). Effect of solar drying on physico‐chemical and antioxidant properties of mango, banana and papaya. National Academy Science Letters, 37(1), 51–57. 10.1007/s40009-013-0196-1

[fsn32324-bib-0002] AOAC (2006). Official methods of analysis Lipids, Fats and Oils Analysis Total Carotenoids—Item 22 (17th ed.). Association of Analytical Communities.

[fsn32324-bib-0003] AOAC International (2007). Official Methods of Analysis of AOAC Inter‐national (18th ed.).

[fsn32324-bib-0004] Babu, A. , Kumaresan, G. , Raj, A. , Velraj, R. , & Reviews, E. (2018). Review of leaf drying: Mechanism and influencing parameters, drying methods, nutrient preservation, and mathematical models. Renewable and Sustainable Energy Reviews, 90, 536–556. 10.1016/j.rser.2018.04.002

[fsn32324-bib-0005] Barragán‐Iglesias, J. , Méndez‐Lagunas, L. L. , & Rodríguez‐Ramírez, J. (2018). Ripeness indexes and physicochemical changes of papaya (*Carica papaya* L. cv. Maradol) during ripening on‐tree. Scientia Horticulturae, 236, 272–278. 10.1016/j.scienta.2017.12.012

[fsn32324-bib-0006] Baye, K. , & Hirvonen, K. (2020). Accelerating progress in improving diets and nutrition in Ethiopia (Vol. 144). Intl Food Policy Res Inst.

[fsn32324-bib-0007] Baye, K. , Hirvonen, K. , Dereje, M. , & Remans, R. (2019). Energy and nutrient production in Ethiopia, 2011–2015: Implications to supporting healthy diets and food systems. PLoS One, 14(3), e0213182. 10.1371/journal.pone.0213182 30861012 PMC6413914

[fsn32324-bib-0008] Baye, K. , & Kennedy, G. (2020). Estimates of dietary quality in infants and young children (6–23 months): Evidence from demographic and health surveys of 49 low‐and middle‐income countries’. Nutrition, 78, 110875. 32653760 10.1016/j.nut.2020.110875

[fsn32324-bib-0009] Bernaert, N. , Van Droogenbroeck, B. , Van Pamel, E. , & De Ruyck, H. (2019). Innovative refractance window drying technology to keep nutrient value during processing. Trends in Food Science & Technology, 84, 22–24. 10.1016/j.tifs.2018.07.029

[fsn32324-bib-0010] Camaschella, C. (2015). Iron‐deficiency anemia. New England Journal of Medicine, 372(19), 1832–1843. 10.1056/NEJMra1401038 25946282

[fsn32324-bib-0011] Eggersdorfer, M. , & Wyss, A. (2018). Carotenoids in human nutrition and health. Archives of Biochemistry and Biophysics, 652, 18–26. 10.1016/j.abb.2018.06.001 29885291

[fsn32324-bib-0012] Guiamba, I. , Ahrné, L. , Khan, M. A. , & Svanberg, U. (2016). Retention of β‐carotene and vitamin C in dried mango osmotically pretreated with osmotic solutions containing calcium or ascorbic acid. Food and Bioproducts Processing, 98, 320–326. 10.1016/j.fbp.2016.02.010

[fsn32324-bib-0013] Herforth, A. , Arimond, M. , Álvarez‐Sánchez, C. , Coates, J. , Christianson, K. , & Muehlhoff, E. (2019). A global review of food‐based dietary guidelines. Advances in Nutrition, 10(4), 590–605. 10.1093/advances/nmy130 31041447 PMC6628851

[fsn32324-bib-0014] Kendall, P. , & Sofos, J. (2007). Drying fruits. Fact Sheet (Colorado State University. Extension). Food and Nutrition Series; No. 9.309.

[fsn32324-bib-0015] Labuza, T. , & Rahman, M. (2007). Water activity and food preservation. Handbook of Food Preservation, 447–476.

[fsn32324-bib-0016] Laillou, A. , Baye, K. , Zelalem, Z. , & Chitekwe, S. (2020). Vitamin A supplementation and estimated number of averted child deaths in Ethiopia: 15 years in practice (2005–2019). Maternal and Child Nutrition. 10.1111/mcn.13132 PMC8189216 33336556

[fsn32324-bib-0017] Lutter, C. K. , & Dewey, K. G. (2003). Proposed nutrient composition for fortified complementary foods. The Journal of Nutrition, 133(9), 3011S–3020S. 10.1093/jn/133.9.3011S 12949402

[fsn32324-bib-0018] Marques, L. G. , Silveira, A. M. , & Freire, J. T. (2006). Freeze‐drying characteristics of tropical fruits. Drying Technology, 24(4), 457–463. 10.1080/07373930600611919

[fsn32324-bib-0019] Melaku, Y. A. , Temesgen, A. M. , Deribew, A. , Tessema, G. A. , Deribe, K. , Sahle, B. W. , Abera, S. F. , Bekele, T. , Lemma, F. , Amare, A. T. , Seid, O. , Endris, K. , Hiruye, A. , Worku, A. , Adams, R. , Taylor, A. W. , Gill, T. K. , Shi, Z. , Afshin, A. , & Forouzanfar, M. H. (2016). The impact of dietary risk factors on the burden of non‐communicable diseases in Ethiopia: Findings from the Global Burden of Disease study 2013. International Journal of Behavioral Nutrition and Physical Activity, 13(1), 122. 10.1186/s12966-016-0447-x PMC5159959 27978839

[fsn32324-bib-0020] Miller, V. , Yusuf, S. , Chow, C. K. , Dehghan, M. , Corsi, D. J. , Lock, K. , Popkin, B. , Rangarajan, S. , Khatib, R. , Lear, S. A. , Mony, P. , Kaur, M. , Mohan, V. , Vijayakumar, K. , Gupta, R. , Kruger, A. , Tsolekile, L. , Mohammadifard, N. , Rahman, O. , … Mente, A. (2016). Availability, affordability, and consumption of fruits and vegetables in 18 countries across income levels: Findings from the Prospective Urban Rural Epidemiology (PURE) study. The Lancet Global Health, 4(10), e695–e703. 10.1016/S2214-109X(16)30186-3 27567348

[fsn32324-bib-0021] Mohammed, S. , Edna, M. , & Siraj, K. (2020). The effect of traditional and improved solar drying methods on the sensory quality and nutritional composition of fruits: A case of mangoes and pineapples. Heliyon, 6, e04163. 32577561 10.1016/j.heliyon.2020.e04163 PMC7305395

[fsn32324-bib-0022] Moses, J. , Norton, T. , Alagusundaram, K. , & Tiwari, B. (2014). Novel drying techniques for the food industry. Food Engineering Reviews, 6(3), 43–55. 10.1007/s12393-014-9078-7

[fsn32324-bib-0023] Osae, R. , Essilfie, G. , Alolga, R. N. , Akaba, S. , Song, X. , Owusu‐Ansah, P. , & Zhou, C. (2020). Application of non‐thermal pretreatment techniques on agricultural products prior to drying: A review. Journal of the Science of Food and Agriculture, 100(6), 2585–2599. 10.1002/jsfa.10284 31975406

[fsn32324-bib-0024] Pradeilles, R. , Baye, K. , & Holdsworth, M. (2019). Addressing malnutrition in low‐and middle‐income countries with double‐duty actions. Proceedings of the Nutrition Society, 78(3). 10.1017/S0029665118002616 30378510

[fsn32324-bib-0025] Quettier‐Deleu, C. , Gressier, B. , Vasseur, J. , Dine, T. , Brunet, C. , Luyckx, M. , Cazin, M. , Cazin, J.‐C. , Bailleul, F. , & Trotin, F. (2000). Phenolic compounds and antioxidant activities of buckwheat (*Fagopyrum esculentum* Moench) hulls and flour. Journal of Ethnopharmacology, 72(1–2), 35–42. 10.1016/S0378-8741(00)00196-3 10967451

[fsn32324-bib-0026] Raghavi, L. , Moses, J. , & Anandharamakrishnan, C. (2018). Refractance window drying of foods: A review. Journal of Food Engineering, 222, 267–275. 10.1016/j.jfoodeng.2017.11.032

[fsn32324-bib-0027] Ratti, C. (2001). Hot air and freeze‐drying of high‐value foods: A review. Journal of Food Engineering, 49(4), 311–319. 10.1016/S0260-8774(00)00228-4

[fsn32324-bib-0028] Rodriguez‐Amaya, D. B. , & Kimura, M. (2004). HarvestPlus handbook for carotenoid analysis (Vol. 2). International Food Policy Research Institute (IFPRI) Washington.

[fsn32324-bib-0029] Ruel, M. T. , Minot, N. , & Smith, L. (2005). Patterns and determinants of fruit and vegetable consumption in sub‐Saharan Africa: A multicountry comparison. WHO Geneva.

[fsn32324-bib-0030] Saini, R. , Shetty, N. , Prakash, M. , & Giridhar, P. (2014). Effect of dehydration methods on retention of carotenoids, tocopherols, ascorbic acid and antioxidant activity in *Moringa oleifera* leaves and preparation of a RTE product. Journal of Food Science and Technology, 51(9), 2176–2182. 10.1007/s13197-014-1264-3 25190880 PMC4152524

[fsn32324-bib-0031] Shende, D. , & Datta, A. K. (2019). Refractance window drying of fruits and vegetables: A review. Journal of the Science of Food and Agriculture, 99(4), 1449–1456. 10.1002/jsfa.9356 30207393

[fsn32324-bib-0032] Singh, P. , Shrivastava, V. , & Kumar, A. (2018). Recent developments in greenhouse solar drying: A review. Renewable and Sustainable Energy Reviews, 82, 3250–3262. 10.1016/j.rser.2017.10.020

[fsn32324-bib-0033] Singleton, V. L. , Orthofer, R. , & Lamuela‐Raventós, R. M. (1999). [14] Analysis of total phenols and other oxidation substrates and antioxidants by means of folin‐ciocalteu reagent. In Methods in enzymology (Vol. 299, pp. 152–178). Elsevier.

[fsn32324-bib-0034] SOFI (2020). The State of Food Security and Nutrition in the World 2020. Transforming food systems for affordable healthy diets. FAO (Food and Agriculture Organization). 10.4060/ca9692en

[fsn32324-bib-0035] Udomkun, P. , Nagle, M. , Argyropoulos, D. , Mahayothee, B. , Latif, S. , & Müller, J. (2016). Compositional and functional dynamics of dried papaya as affected by storage time and packaging material. Food Chemistry, 196, 712–719. 10.1016/j.foodchem.2015.09.103 26593545

[fsn32324-bib-0036] WHO (2004). Vitamin and mineral requirements in human nutrition. World Health Organization.

[fsn32324-bib-0037] Worku, I. H. , Dereje, M. , Minten, B. , & Hirvonen, K. (2017). Diet transformation in Africa: The case of Ethiopia. Agricultural Economics., 48(S1), 73–86. 10.1111/agec.12387

[fsn32324-bib-0038] World Health Organization (WHO) . (2017). The double burden of malnutrition. Policy brief. World Health Organization.

[fsn32324-bib-0039] Yap, J. Y. , Hii, C. L. , Ong, S. P. , Lim, K. H. , Abas, F. , & Pin, K. Y. (2020). Effects of drying on total polyphenols content and antioxidant properties of Carica papaya leaves. Journal of the Science of Food and Agriculture, 100(7), 2932–2937. 32031257 10.1002/jsfa.10320

