# Supplementary material for: Effects of different drying methods and ascorbic acid pretreatment on carotenoids and polyphenols of papaya fruit in Ethiopia
Source: Food Sci Nutr. 2021 May 4;9(6):3346–53. doi: 10.1002/fsn3.2324 (PMC8194739; doi:10.1002/fsn3.2324)
Supplement: Supplementary file 1 — Fig S1‐S2 [file FSN3-9-3346-s001.docx]

**SUPPLEMENT**

**
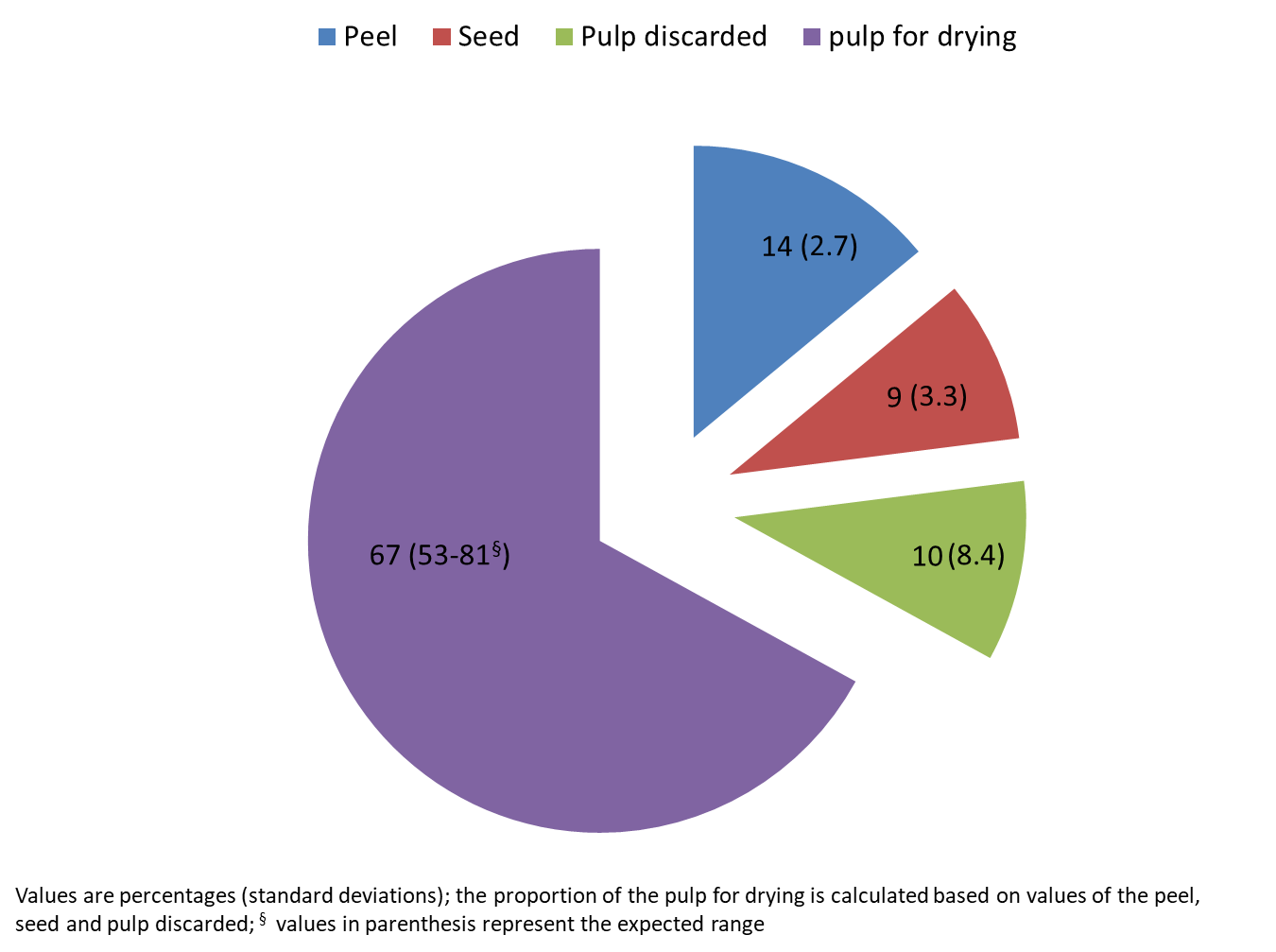
**

**Fig S1** Proportion of peel, seed, and pulp in hand-prepared fresh papaya

Values are percentages (standard deviations); the proportion of the pulp for drying is calculated based on values of the peel, seed and pulp discarded; ^§^ values in parenthesis represent the expected range


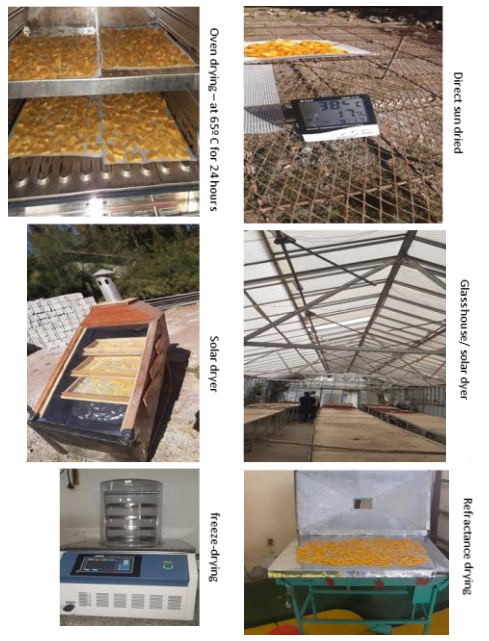


**Fig S2** Picture of drying techniques used in the study
